# Effect οf Genotype and Growing Year on the Nutritional, Phytochemical, and Antioxidant Properties of Industrial Hemp (*Cannabis sativa* L.) Seeds

**DOI:** 10.3390/antiox8100491

**Published:** 2019-10-17

**Authors:** Maria Irakli, Eleni Tsaliki, Apostolos Kalivas, Fotios Kleisiaris, Eirini Sarrou, Catherine M Cook

**Affiliations:** Institute of Plant Breeding & Genetic Resources, Hellenic Agricultural Organization—Demeter, 57001 Thermi Thessaloniki, Greece; tsaliki@ipgrb.gr (E.T.); kalyvas@ipgrb.gr (A.K.); cerealtech@cerealinstitute.gr (F.K.); sarrou@ipgrb.gr (E.S.); cook@nagref.gr (C.M.C.)

**Keywords:** hemp seed (*Cannabis sativa* L.), nutritional components, fatty acid profile, phytochemicals, lignanamides, antioxidant, genotype, growing year

## Abstract

*Cannabis sativa* L. seeds have been an important source of protein, oil, and dietary fiber for human and animals. Currently, there is a growing interest in the commercial products of these seeds, which are recognized as a legitimate source of medicaments, cosmeceuticals, and nutraceuticals. The objective of this study was to investigate the nutritional, phytochemical composition, and antioxidant properties of seeds from seven hemp cultivars grown in Greece for three consecutive years. All the measured parameters strongly varied under the influence of growing year and genotype. In particular, protein, oil, and carbohydrates’ content of hemp seeds as well as fatty acids’ composition were mainly affected by genotype, whereas the growing year had a major effect on phytochemical components and antioxidant activity, which was determined by the 2,2′-azino-bis (3-ethylbenzthiazoline sulfonate) (ABTS) and ferric-reducing antioxidant power (FRAP) assays. Moreover, a predominant effect of the year was observed for phenolic profiles as determined by high-performance liquid chromatography and total carotenoids’ content. This study suggests that hemp seeds could be a promising food crop as a result of their high nutritive traits and antioxidant potential. A comparison of the studied cultivars, showed that Finola seeds had the highest oil and protein contents and, thus, appeared to be the most promising cultivar for cultivation in Greece.

## 1. Introduction

Industrial hemp (*Cannabis sativa* L.) is an ancient crop of Asian origin that has been traditionally cultivated in many regions of Europe for use as a textile, until the early twentieth century, after which its cultivation declined in most Western countries [1]. Hemp is cultivated as a multi-purpose crop in a plethora of many agro-industrial fields such as agriculture, textiles, bio-composites, papermaking, construction, biofuel, functional foods, personal care, and cosmetics [2]. Fiber, essential oils, and seeds are valuable products derived from different parts of the plant. Recently, hemp has been shown to be a valuable option in the production of sustainable bioenergy [3]. 

Hemp seeds are mainly used as animal feed, but their products (oil, meal, flour, and protein powder) are gaining in the market with a growing interest in their usage for human nutrition. They can be served as a new, natural, and excellent source of nutrients, containing all the essential amino acids and fatty acids necessary to maintain a healthy human life [3]. They contain 25–35% oil, 20–25% protein, 20–30% carbohydrates, and 10–15% insoluble fiber, vitamins, and minerals [4]. In particular, the hemp seed oil is high in polyunsaturated fatty acids, with an ideal ratio (2.5–3:1) of α-linolenic acid (ω-6) and linoleic acid (ω-3) for human nutrition [5,6]. These factors should give hemp seeds a strong market value and make it likely that the primary end use would be in human food and nutritional supplements [7].

In addition to its nutritional value, hemp seed is also rich in natural antioxidants such as phenolic compounds, tocopherols, and phytosterols [3], which may play a role in reducing the risk of chronic diseases, including cancer, neurodegenerative diseases, lipid metabolism, cardiovascular health, immunomodulatory effects, dermatological diseases, and gastrointestinal disorders [8,9]. A wide range of polyphenols have been identified in hemp, especially flavonoids such as flavanones, flavanols, flavonols, and isoflavones [10,11], whereas the predominant phenolics in hemp seeds are simple and complex lignanamides, which display interesting and diverse biological activities, including feeding deterrent activity, insecticidal effects, and anti-inflammatory activity [12]. Other bioactive compounds of the hempseed oily fraction are tocopherols, which act as antioxidants and prevent the oxidation of unsaturated fatty acids [5]. 

From an agronomical point of view, hemp is considered to be a high-yielding crop that requires few technical inputs with very high resistance to drought and pests. This is a well-developed root system preventing soil erosion and lower water requirement with respect to other crops [3]. In 2016, hemp cultivation was progressively authorized in Greece, under strict control and monitoring by the Ministry of Rural Development and Food. According to EU legislation (No 809/2014 of 17 July 2014), production of hemp is permitted if the content of the principal psychoactive constituent, Δ-9-tetrahydrocannabinol (THC), is less than 0.2%. Some studies have shown that different factors, including the genotype and environment, may have large effects on both fiber and seed production [13]. Comparative studies between the seed composition of industrial hemp cultivars of genetic variation are available [14,15,16]. The effect of the genotype and environment on the main seed characteristics have been investigated [6,17], whereas comparative studies concerning the influence of the genotype and environment on the nutritional and antioxidant properties of hemp seed are scarce.

This study was, therefore, designed to evaluate the effects of genotype and growing year on the nutritional and phytochemical profile, as well as the antioxidant activity of seeds of seven industrial hemp cultivars cultivated in Greece in three consecutive years, in order to promote the use of this crop in the Mediterranean food chain.

## 2. Materials and Methods

### 2.1. Chemicals

2,2′-azino-bis (3-ethylbenzthiazoline sulfonate) (ABTS), 2,4,6-tripyridyl-s-triazine (TPTZ), 6-hydroxy-2,5,7,8-tetramethylchroman-2-carboxylic acid (Trolox), and Folin–Ciocalteu reagent were purchased from Sigma-Aldrich (Steinheim, Germany). High purity analytical standards (>98%) of phenolic acids were supplied by Sigma-Aldrich (Steinheim, Germany), while tocopherol isomers were bought from Supelco (Belefonte, PA, USA). Lutein was purchased from Applichem (Darmstadt, Germany). β-carotene was obtained from Fluka (Buchs, Switzerland) and zeaxanthin was from Extrasynthese (Genay, France). All other reagents obtained from Chem-Lab (Zedelgem, Belgium) were of an analytical or high performance liquid chromatography (HPLC) grade.

### 2.2. Plant Material and Agricultural Conditions

The seeds of seven industrial hemp cultivars, which are Santhica 27 (France), Fedora 32 (France), Felina 32 (France), Futura 75 (France), Tygra (Poland), Bialobrzeskie (Poland), and Finola (Finland), were harvested in three successive growing years (2016, 2017, and 2018). The above cultivars have been selected on the basis of their Δ-9-tetrahydrocannabinol (THC) content as required by EC regulation (No 809/2014 of 17 July 2014). Furthermore, the monoecious cultivars have dual use (fiber and seed production) and are well adapted to Greek climatic conditions. The dioecious variety Finola is the main cultivated variety for seed production in Greece and it covers half of the hemp cultivation area each year. The main characteristics of the cultivars are reported in Table 1. In 2016, Fedora and Finola were not cultivated. Field trials were carried out at the experimental station of the Hellenic Agricultural Organization—Demeter, Institute of Plant Breeding and Genetic Resources (Thermi, Thessaloniki, Greece, latitude 40°32′49.63″ N, longitude 23°01′10.81″ E) in a silty loam soil, with 32% silt, 18.2% clay, 18.2% sand, 1.6% organic matter, and pH of 7.6–7.9. Some climatic parameters of the cultivated study site are given in Table 2, while wheat was the main cultivated crop in the experimental field, in previous years.

Hemp seeds were sown by hand at a rate of 50 Kg ha^−1^ on May 18, April 3, and April 12 in 2016, 2017, and 2018, respectively, following a randomized block experimental design with four parcel repetitions. Each plot had a size of 20 m^2^ with a distance of 17 cm between rows. No pesticides were used. Before sowing, basic fertilization (11-15-15) was applied at a rate of 200 kg ha^−1^. During the summer, the plants were irrigated when necessary.

Inflorescences from 100 plants from each plot were randomly collected at seed maturity when the stems had shed their leaves and more than 70% of seeds were hard, from August to September. After harvesting, seeds were separated from the leaves and stems using a 2-mm sieve and a zig-zag aspirator and gravity separator (ZZ SERIE, Selecta). Seeds were ground using a Retsch ZM 1000 laboratory mill in order to pass through a 0.5-mm sieve and, then, were stored at 4 °C until their analyses. In order to ensure that the industrial hemp cultivars studied are suitable for agricultural production, the Δ9-tetrahydrocannabinol (THC) content of inflorescences was determined according to the method described in EC regulation 809/2014 [18]. 

### 2.3. Chemical Composition

Seed proximate composition (moisture, crude protein, crude fat, and total ash) was determined using the Methods of Association of Official Agricultural Chemists (AOAC) International [19]. The carbohydrate composition was determined by the difference.

### 2.4. Fatty Acid Composition

The fatty acid composition of the hemp seed oils extracted with ether was determined using gas chromatography with flame ionization detection (Model Varian CP-3800, Middelburg, The Netherlands) based on the AOAC 996.06 method [20]. Fatty acid methyl esters were identified by comparison of their retention times with those of external standards (Supelco 37 Component FAME Mix) and the amount of individual fatty acids was expressed as percentages (%) of the total fatty acids determined. Each sample was analyzed in triplicate.

### 2.5. Tocopherols and Carotenoids Composition

Tocopherols and carotenoid content in the hemp seed samples were determined, according to a modified method of Irakli et al. [21]. A total of 0.5g of hemp seed flour was sonicated with ethanol (10mL) and the extract was collected after centrifugation at 1500× *g* for 10 min. The same procedure was repeated twice. Two mL of the collected supernatants was evaporated under the flow of nitrogen. The remaining residue was re-dissolved in 400 μL of the HPLC initial mobile phase and filtered through a 0.22-μm Nylon membrane prior to HPLC analysis. The HPLC analysis of tocopherols and carotenoids was performed using an HPLC system (Agilent Technologies, 1200 series, Urdorf, Switzerland) equipped with a YMC C_30_ column (250 × 4.6 mm id, 3 μm, MZ Analysentechnik, Mainz, Germany). The mobile phase consisted of acetonitrile (A), methanol (B), and dichloromethane (C) with a gradient system: 0 min, 85% A and 15% B, 5 min, 65% A, and 35% B, 10 min, 10% A, 85% B, and 5% C, 15 min, 30% A, 40% B, and 30% C. The flow rate was 1.5 mL min^−1^ and the injection volume was 20 μL. Tocopherol isomers (α-Τ, β-Τ, γ-Τ, and δ-Τ) were detected by a fluorescence detector with excitation and emission wavelengths at 290 and 320 nm, respectively, whereas carotenoid compounds (lutein, zeaxanthin, and β-carotene) were detected at 450 nm. External calibration curves were constructed using standard solutions and the results were expressed as mg per 100 g of hemp seed flour (mg 100 g^−1^).

### 2.6. Polyphenol Extraction 

Hemp seed flour (10 g) was extracted with hexane in a soxhlet apparatus to remove the oil from the seeds. The residual hemp cake was used for the extraction of phenolic compounds with 80% aqueous methanol (*v/v*) for 30 min at 65 °C using an ultrasonic bath (frequency 37 kHz, model FB 15051, Thermo Fisher Scientific Inc., Loughborough, England). The extract was then centrifuged at 10,000× g for 10 min at 4 °C and the clear supernatant was stored at −20 °C until analysis. 

### 2.7. Total Phenolic Content

The total phenolic content (TPC) of extracts was determined by the Folin-Ciocalteu reagent method [22]. Gallic acid was used as standard compound and polyphenol content was expressed as milligrams of gallic acid equivalents (GAE) per 100 g of seed (mg GAE 100 g^−1^).

### 2.8. Phenolic Acid Composition

The phenolic extracts (see Section 2.6) obtained for each hemp cultivar were used to determine the polyphenol profile by High performance liquid chromatography with diode array detector (HPLC-DAD). The HPLC-analysis was performed using an Agilent Technologies HPLC (1200 series, Urdorf, Switzerland) system equipped with a Nucleosil 100 C_18_ column (250 mm × 4.6 mm, i.d. 5 μm). The mobile phase consisted of 1% (*v/v*) aqueous acetic acid and methanol and was based on a previously described method [23]. Each phenolic compound in the hemp seed extract was identified by comparison of their retention times to those of external standards with the exception of N-*trans*-caffeoyltyramine and cannabisin A due to the lack of available commercial standards. The quantification was based on standard curves generated by the external standard method. N-*trans*-caffeoyltyramine and cannabisin A were expressed in *trans*-cinnamic acid equivalents.

### 2.9. Antioxidant Activity Assays

Two assays were employed to determine the antioxidant activities of the hemp seed extracts: ABTS assay [24] and ferric-reducing antioxidant power (FRAP) [25]. Trolox was used as the standard compound for calibration curves and the results were expressed in mg of trolox equivalents (TE) per 100 g of hemp seed (mg TE 100 g^−1^). 

### 2.10. Statistical Analysis

Values were reported as the mean ± standard deviation of triplicate measurements. All parameters were subjected by two-way analysis of variance (ANOVA) to evaluate the effect of genotype, year, and their interaction using a fixed model (SPSS Inc. Chicago, IL, USA). The differences between mean values were evaluated by using Tukey’s test at the level of significance *p* < 0.05. The Pearson’s Product Moment correlation was also used in order to determine possible correlations between the constituents determined from the phytochemical analysis.

## 3. Results and Discussion

All of the seven industrial hemp cultivars grown in this study contained < 0.2% (*w/w*) Δ-9-tetrahydrocannabinol (THC) (data not shown), which is the permitted level for cultivation. According to EU legislation, which is implemented by the Greek Ministry of Rural Development and Food, production of hemp is permitted if the content of the principal psychoactive constituent, Δ-9-tetrahydrocannabinol (THC), is less than 0.2%. 

Recently, an upward trend in hemp production has been observed in Greece with about 2.6, 30.0, and 108.5 hectares (ha) being cultivated in 2016, 2017, and 2018, respectively. In the current study, we have focused on hemp seeds, which are rich in nutritional and phytochemical components, and are regarded as nutraceuticals.

### 3.1. Effect of Genotype and Growing Year on the Nutritional Components

**Figure 1:**
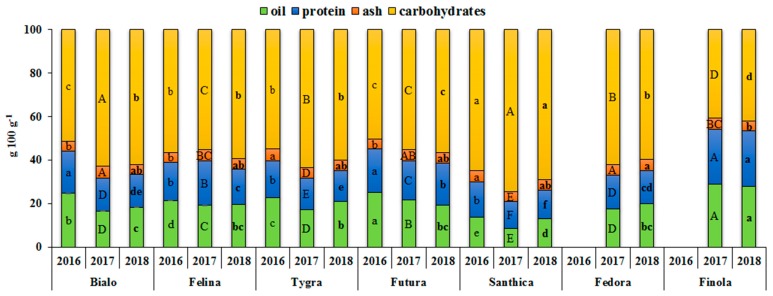
Nutritional profile showing the oil, protein, ash, and carbohydrate contents (g 100 g^−1^ seed) of hemp seeds from the seven industrial hemp cultivars cultivated in this study and for three growing seasons (2016, 2017, and 2018). For each year, means within columns in the same color followed by the same letter (lower case, capitals, and lower case in bold for 2016, 2017, and 2018, respectively) are not significantly different (*p* > 0.05).

The nutritional composition of seeds of the seven hemp cultivars cultivated in Greece, in three consecutive years (2016, 2017, and 2018) is given in Figure 1. The hemp seeds’ chemical compositions differed between the seven cultivars with contents (g 100 g^−1^ hemp seed flour) ranging from 8.5% to 29.2% for oil, from 12.2% to 25.4% for protein, from 4.4% to 5.3% for ash, and from 40.8% to 74.5% for carbohydrates. On average, for all cultivars and taking into account that two were not cultivated in 2016, the hemp seeds contained approximately 20.0% oil and 17.4% protein. Finola had the highest oil and protein content, while Santhica had the lowest for the three years. On the contrary, Santhica had the highest carbohydrate content with a mean value of 57.8% and Finola had the lowest. On the other hand, the ash content had a mean value of 4.8% for all cultivars and years cultivated.

Analysis of variance (ANOVA) revealed significant differences (*p* < 0.001) in the oil, protein, ash, and carbohydrates’ contents among seven cultivars analyzed. The genotypic variance accounted for 58.8%, 52.4%, 9.6%, and 57.7% of the total variation, respectively (Table 3). It is clear that the most important variance component for the protein, oil, and carbohydrate contents measured in the present study was the genotype (G), when compared with the much lower effects of growing year (Y) and their interaction (GxY). In contrast, the hemp genotype had a minor effect on ash content. The effects of year were significant (*p* < 0.001), with oil and protein contents slightly higher in seeds harvested in 2016 when compared to 2017 and 2018 (Table 3, Figure 1), in contrast to the ash and carbohydrate contents. In addition, the GxY was also significant (*p* < 0.001), even though all traits, except for the ash content, contributed to less than 10% of the total variance (Table 3).

In the current study, the mean oil and protein content of seeds collected from Finola cultivar (28.6% and 25.2%, respectively) were in general agreement with previously published data for this cultivar [4,10,15,26]. However, for the other cultivars grown in Greece, such as Fedora and Felina, in some studies, the values were lower than those for plants grown in Italy [16,27], while, in other reports, similar oil values were presented for the same varieties grown in Italy [6]. This indicates that geography, climatic conditions, and local agronomic factors may affect hemp seed composition. Lastly, these results indicate that, out of the hemp cultivars tested, Finola, a cultivar mainly grown for seed production as a good source of oil and protein [4,26], appeared to be the most adapted to Greek environmental conditions in terms of the nutritional composition of its seeds.

### 3.2. Effect of Genotype and Growing Year on the Fatty Acid Profile

The fatty acid composition of the hemp seed oils is shown in Table 4. The principal saturated fatty acids (SFA) in the hemp seeds from the seven cultivars were palmitic acid ranging from 7.1% (Finola) to 9.1% (Santhica) and stearic acid, ranging from 2.1% (Finola) to 2.8% (Tygra and Futura), whereas minor SFA were arahidic, behenic, and lignoceric acids. The major mono-unsaturated fatty acid (MUFA) was oleic acid ranging from 10.3% (Finola) to 17.9% (Futura), whereas trace amounts of eicosenoic acid were found. With respect to polyunsaturated fatty acids (PUFA), hemp seed cultivars contained an average of 53.4% linoleic, 12.1% α-linolenic, and 3.0% γ-linolenic acids. Specifically, linoleic acid ranged from 51.6% (Tygra) to 54.2% (Felina and Santhica), while α-linolenic ranged from 10.5% (Bialo) to 15.3% (Finola) and γ-linolenic acid from 1.9% (Futura) to 5.0% (Finola). It is noteworthy that, of the hemp seed cultivars, Finola showed the highest content of γ-linolenic and α-linolenic acids and the lowest oleic acid and SFA, which include palmitic and stearic acid content. This is in agreement with results reported by other researchers [6,15,27]. 

The average ω-6/ω-3 ratio was 4.8, which indicates a good nutritional index for the hemp seeds studied and is close to values reported in the literature [4,16,27]. In highly developed countries, people’s diets contain large amount of ω-6 acids, so it is important for human health to reduce the ω-6/ω-3 ratio in the diet, by including plant oils with a low ratio of ω-6/ω-3, such as hemp oil. Although the ideal ratio of ω-6/ω-3 has been established as 3:1 to 5:1, there are differing opinions in the literature [28,29]. Among the genotypes, Finola showed the lowest ω-6/ω-3 ratio with a mean value of 3.9, while the other hemp cultivars studied had the highest ratio, with a mean value of 4.9. These results are in accordance with those of Da Porto et al. [30]. Generally, the studied hemp seed oils were characterized by a high PUFA content and a low MUFA and SFA content. The contents of PUFA on average corresponded to 70.0% of the total fatty acids identified, while the MUFA and SFA found in the hemp seed oils accounted for 15.9% and 12.6% of the total fatty acids identified, respectively.

ANOVA results showed that the fatty acid composition of hemp seeds from the seven cultivars was drastically affected by G, with mean values of genotypic variance, accounting for approximately 80% of the total variance (Table 3). Among the fatty acids identified, α-linolenic acid was most affected by G (99.6%), which is followed by oleic (91.2%) and palmitic acids (86.2%), whereas linoleic acid was least affected (42%). Moreover, the effects of Y and GxY were also significant (*p* < 0.001), even though all traits, except for linoleic acid, contributed to less than 26% of the total variance. It is worth noting that the ω-6/ω-3 ratio was not significantly (*p* > 0.05) influenced by the Y, whereas the GxY was significant, but with a low contribution (13.4%) to total variance.

### 3.3. Effect of Genotype and Growing Year on the Tocopherols and Carotenoid Content

All the hemp seed samples analyzed in this study contained primarily γ-tocopherol and lesser quantities of α-tocopherols and δ-tocopherols (Table 5). On average, γ-tocopherol concentration of the hemp seed analyzed from all cultivars was 7.0 mg 100 g^−1^ seed flour, which was approximately 10 times higher than that of δ-tocopherol (0.8 mg 100 g^−1^ seed flour), while α-tocopherol was only detected in trace amounts. The highest γ-tocopherol content was detected in Finola (11.3 mg 100 g^−1^ seed flour) and the lowest was detected in Santhica (4.6 mg 100 g^−1^ seed flour), whereas the highest δ-tocopherol contents was found in Finola (1.3 mg 100 g^−1^ seed flour) and the lowest was found in Fedora, Santhica, Felina, and Bialo (0.6 mg 100 g^−1^ seed flour). These ranges of tocopherol concentrations detected in hemp seeds in this study were much lower than those reported for seeds from hemp cultivars grown in Canada by Vonapartis et al. [15], but much higher than those reported for cultivars grown in Italy by Galasso et al. [27]. This discrepancy may be due to different extraction protocols. Tocopherols, as natural antioxidants, can prevent the oxidation of oil and also lower the risk of cardiovascular diseases, cancer, and age-related macular degeneration, when included in the diet [31].

The analysis of variance showed that the G as well as Y and GxY had significantly influenced the tocopherol contents. However, the Y accounted for the greatest variability, at more than 70% (Table 3). Similarly, Galasso et al. [27] also found a high variability (*p* < 0.01) in tocopherol content among the genotypes analyzed.

Carotenoids were another group of bioactive compounds analyzed. They are important for plant growth and photosynthesis and are essential components of human diets, as β-carotene is a precursor of vitamin A and this is beneficial to vision [32]. The hemp seed for the seven cultivars contained three main carotenoids known as lutein, zeaxanthin, and β-carotene. Lutein was the primary carotenoid ranging from 1.5 (Futura) to 3.4 mg 100 g^−1^ (Santhica), with a mean value of 2.1 mg 100 g^−1^ for all cultivars, while zeaxanthin content ranged from 0.2 (Tygra, Finola, and Fedora) to 0.5 mg 100 g^−1^ (Futura) and β-carotene content from 0.2 (Bialo, Futura, and Finola) to 0.8 mg 100 g^−1^ (Tygra) (Table 5). There are few reports on the content of carotenoids in hemp seeds in order to compare our results, with most publications referring to hemp seed oils. The total carotene content reported by Aladic et al. [33] was 3.14 mg 100 g^−1^ and 12.50 mg 100 g^−1^ in hemp seed oil produced by a cold pressing process and supercritical fluid extraction, respectively. 

In the present study, all carotenoid contents were affected (*p* < 0.001) by both G and Y as well as by GxY (Table 3). The Y interacted primarily with zeaxanthin content, while G affected lutein and β-carotene contents. The main factors contributing to total carotenoid contents were the G (54.4%) and the GxY (38.4%), while a slight but significant effect of the Y was observed (7.4%). These finding are in agreement with previous investigations reporting the genetic determination of yellow pigment content in wheat grains [34].

### 3.4. Effect of Genotype and Cultivation Year on the Phenolics Profile and Antioxidant Activity

**Figure 2:**
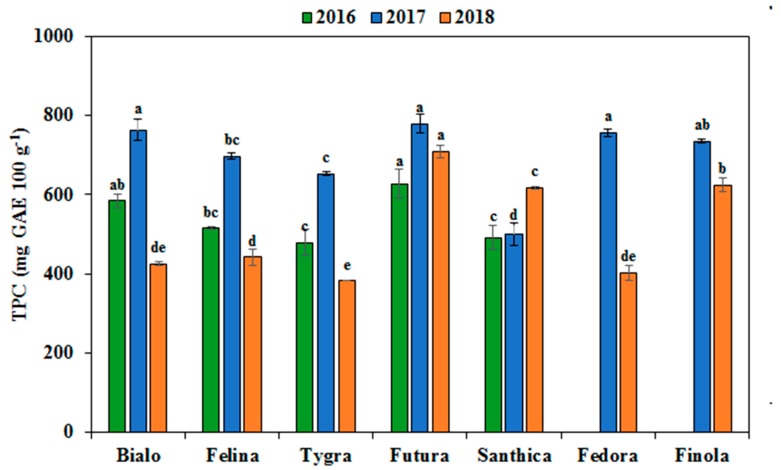
Total phenolic content (TPC) of hemp seeds from the seven industrial hemp cultivars cultivated in this study for three growing seasons (2016, 2017, and 2018). For each year, means within the column in the same color followed by the same letter are not significantly different (*p* > 0.05).

Phenolic compounds play an important role in human health due to their role in the prevention of cancer and several chronic diseases, by acting as radical scavengers [35]. As shown in Figure 2, the total phenolic content (TPC) of hemp seed from the seven cultivars ranged from 381.8 (Tygra) to 779.8 mg 100 g^−1^ (Futura), with a mean value of 588.8 mg 100 g^−1^. The TPC ranged from 478.4 to 627.3 mg 100 g^−1^ in 2016, from 500.0 to 780.0 mg 100 g^−1^ in 2017 and from 381.8 to 709.1 mg 100 g^−1^ in 2018. Higher TPC were determined in 2017 for most of the genotypes, except for Santhica, which had the highest TPC in 2018. Similar ranges for TPC in hemp seeds have been reported by other researchers [27,36,37]. However, Vonapartis et al. [15] and Pagnani et al. [38] reported higher values, likely due to agronomical or environmental factors. In the present study, Futura had the highest TPC in all years, whereas Tygra and Santhica (with the exception of Santhica in 2018) had the lowest TPC.

ANOVA indicated that TPC was affected significantly (*p* < 0.001) by both G and Y, as well as by GxY (Table 3). The primary factor contributing to TPC was the Y (58.9%), followed by G (27.3%) and GxY (13.8%). Heimleret et al. [39] found that temperature conditions before harvesting of wheat seeds were the main factor influencing the profile of phenolic compounds and their anti-oxidant activity of in a two-year study. In the present study, the main difference in the environmental conditions between the growing years was the high rainfall in the period in July and August of 2017, during which flowering and grain-filling of the hemp cultivars occurred and only minor temperature differences were observed. These conditions might be responsible for the high values of total phenolics and antioxidant activity in 2017 compared to other years, but further studies are required to explain the exact role of environmental conditions on these two properties.

**Figure 3:**
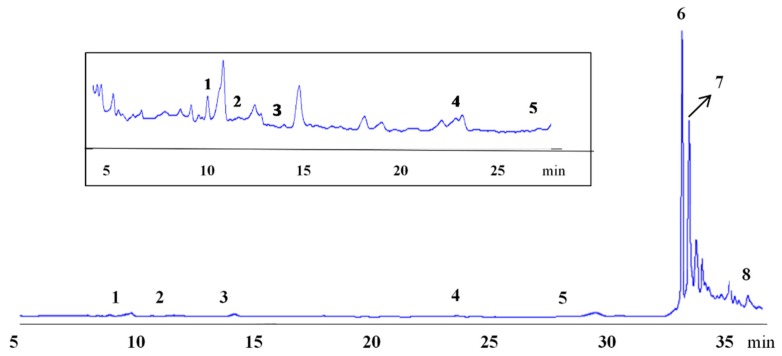
High performance liquid chromatography (HPLC) chromatogram of Felina hempseed phenolic extract at 280 nm. Peaks identification: 1, protocatechuic acid. 2,4-hydroxybenzoic acid. 3, vanillic acid. 4, p-coumaric acid. 5, ferulic acid. 6, N-*trans*-caffeoyl-tyramine. 7, cannabisin A. 8, cinnamic acid.

The reversed phase-HPLC chromatogram obtained for the phenolic extract of hemp seeds from the cultivar Futura, monitored at 280 nm, is shown in Figure 3. The phenolic compounds like N-*trans*-caffeoyl-tyramine (UV λ_max_ in MeOH 220, 294, 318 nm) and cannabisin A (UV λ_max_ in MeOH 257 nm) were identified on the basis of their spectral characteristics, according to literature data [37,40]. 

**Figure 4:**
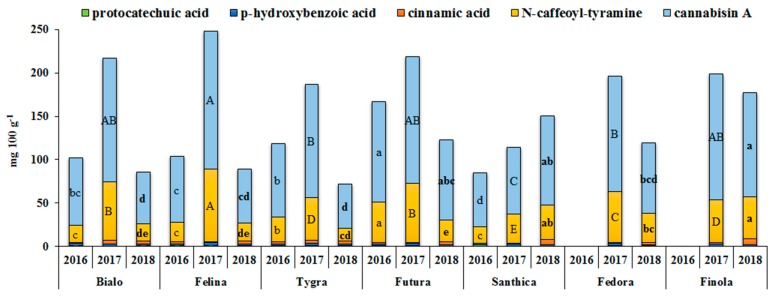
The main phenolic acid components of hemp seeds from the seven industrial hemp cultivars cultivated in this study for three growing seasons (2016, 2017, and 2018). For each year, means within the column in the same color followed by the same letter (lower case, capitals, and lower case in bold for 2016, 2017, and 2018, respectively) are not significantly different (*p* > 0.05) (N-*trans*-caffeoyl-tyramine and cannabisin A are expressed in *trans*-cinnamic acid equivalents).

The main phenolic compounds that have been detected in *Cannabis sativa* fruits, seeds, and roots are lignans, belonging to two main groups known as phenolic amides and lignanamides [40,41]. Lignans show a wide range of health-promoting properties including antioxidant, antiviral, antidiabetic, antitumorigenic, and anti-obesity activities [42]. As shown in Figure 4, the major phenolic amide in all hemp seed extracts was N-*trans*-caffeoyltyramine, ranging from 14.8 (Tygra) to 83.2 mg 100 g^−1^ (Felina), with a mean value of 39.2 mg 100 g^−1^ for all cultivars, whereas the predominant lignanamide was cannabisin A, ranging from 51.1 (Tygra) to 159.1 mg 100 g^−1^ (Felina), with a mean value of 100.9 mg 100 g^−1^ for all cultivars. Apart from those, the other phenolic compounds detected in hemp extracts were protocatechuic acid, p-hydroxybenzoic acid, and cinnamic acid, ranging from 0.4 to 1.6 mg 100 g^−1^, from 1.2 to 3.0 mg 100 g^−1^, and from 0.2 to 7.3 mg 100 g^−1^, respectively. Minor phenolic compounds detected were vanillic acid, p-coumaric acid, and ferulic acid. A similar phenolic profile was also reported by Pojić et al. [43] and Smeriglio et al. [10].

The phenolic profile of hemp seed extracts was affected (*p* < 0.001) by G as well as by Y. However, the variability of Y (>67%) was higher than that of G. Of all hemp cultivars tested, Futura and Finola had the highest cannabisin A content in all cultivation years, whereas the highest content of N-*trans*-caffeoyl-tyramine was detected in Futura, Felina, and Finola for all cultivation years. Most notably in 2017, all the hemp cultivars, except Santhica, contained significantly more cannabisin A and N-*trans*-caffeoyltyramine, which are the predominant phenolic compounds when compared to the other years. 

**Figure 5:**
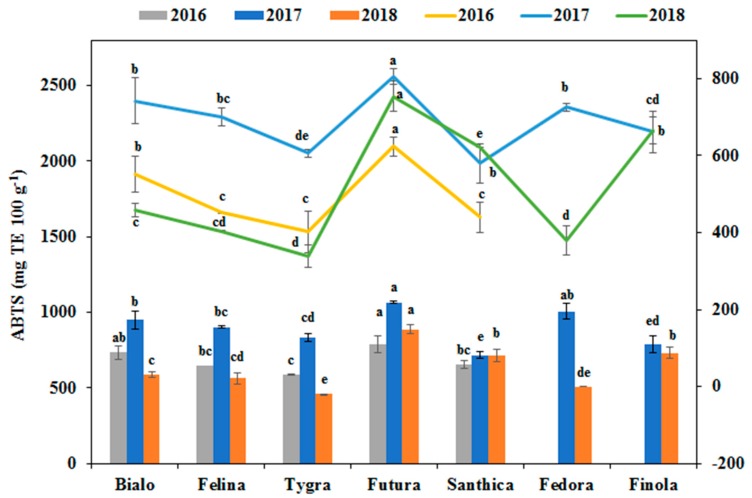
Antioxidant activity of extracts of hemp seeds from the seven industrial hemp cultivars cultivated in this study and for three growing seasons (2016, 2017, and 2018) as measured by 2,2′-azino-bis (3-ethylbenzthiazoline sulfonate) (ABTS) and ferric-reducing antioxidant power (FRAP) assays. For each year, means within the column in the same color or in the same line followed by the same letter are not significantly different (*p* > 0.05).

Similarly, in 2017, higher antioxidant activity as measured by ABTS and FRAP assays were obtained for the hemp cultivars, which was in accordance with the TPC of the extract (Figure 5). These results indicate that the above-mentioned phenolic compounds contribute to the antioxidant potential of a genotype. Both antioxidant assay methods used for analyzing hemp seed extracts revealed that Futura had the highest antioxidant potential among the seven cultivars (*p* < 0.001). Specifically, Futura seed extracts exhibited powerful ABTS• radical-scavenging activity ranging from 458.8 to 1066.3 mg TE 100 g^−1^, with a mean value of 734.2 mg TE 100 g^−1^. Their antioxidant activity was further confirmed by the FRAP assay, with values ranging from 338.4 to 806.8 mg TE 100 g^−1^, with a mean value of 574.5 mg TE 100 g^−1^. 

Significant differences in antioxidant activity among G and Y were observed (*p* < 0.001). Y had a more significant impact on antioxidant activity than G (Table 3). For most of the hemp cultivars, the antioxidant activity (ABTS and FRAP assays) in different years followed the same order: 2017 > 2016 > 2018.

The correlations between the two antioxidant activities (ABTS and FRAP) and TPC, N-*trans*-caffeoyltyramine, cannbisin A, and total tocopherols in all hemp seed extracts for the three years of cultivation was evaluated using Pearson correlation analysis. A significant (*p* < 0.01) positive correlation was observed between the TPC and antioxidant activities (data not shown). Both the ABTS (*r =* 0.945, *p* < 0.01) and FRAP (*r* = 0.946, *p* < 0.01) assays had the greatest correlation with polyphenols, which means that antioxidant indexes are mainly affected by polyphenols, in accordance with previous findings [37,44]. It has been reported that phenolic compounds, such as lignanamides, show high antioxidant activity [12]. In our study, a highly significant linear correlation was exhibited between N-*trans*-caffeoyl-tyramine and ABTS and FRAP values, with *r* = 0.766 and *r* = 0.752, respectively (*p* < 0.01) as well as between cannabisin A with ABTS and FRAP values, with *r* = 0.800 and *r* = 0.811 (*p* < 0.01), respectively. Weak correlations were also found between γ-tocopherol, which is the main lipophilic antioxidant component present in appreciable amounts in hemp seed extracts and antioxidant activity, with *r* = 0.324 (*p* < 0.05) and *r* = 0.352 (*p* < 0.01) for ABTS and FRAP values, respectively. Similarly, other researchers have found no correlation between vitamin E content and antioxidant activity, as evaluated by DPPH (1, 1diphenyl-2-picryl hydrazyl) and ABTS assays in cereal grains [45]. Limited information is available on the bioactive compositions and antioxidant activities of lipophilic compounds from hemp seed extracts. The low levels of carotenoids detected in this study did not correlate with the antioxidant activity measured. Overall, the data clearly indicate that the antioxidant capacity of hemp seed extracts is due, mainly, to phenolic amides and lignanamides rather than to tocopherols.

## 4. Conclusions

In conclusion, the cultivation of industrial hemp in the same site in Greece for three years showed that the nutritional and phytochemical profile and, thereby, the level of the total antioxidant activity in industrial hemp seeds are affected by genotype and growing year. Our results highlight that protein and oil contents, which are the interesting nutritional components of hemp seeds, and the fatty acid composition are mainly affected by genetic factors, in contrast to the total phenolics, tocopherols, and carotenoids contents as well as antioxidant activity, which appear to be mostly affected by the growing year and by the genotype. These findings could help select hemp cultivars as seeds with higher nutritional or antioxidant compounds, which will aid in the identification of potential food applications and nutraceuticals. In addition, it can help breeding programs develop industrial hemp varieties with improved levels of nutrients and antioxidants, which can provide better health and industrial benefits.

## Figures and Tables

**Table 1:** Main characteristics of the seven industrial hemp cultivars cultivated in Greece in the present study.

Genotype	Abbreviations	Origin	Species
Bialobrzeskie	Bialo	Poland	monoecious
Felina 32	Felina	France	monoecious
Tygra	Tygra	Poland	monoecious
Futura 75	Futura	France	monoecious
Santhica 27	Santhica	France	monoecious
Fedora 17	Fedora	France	monoecious
Finola	Finola	Finland	dioecious

**Table 2:** Monthly mean maximum temperature (T_max_), minimum temperature (T_min_), and total rainfall during the growing seasons of industrial hemp at Thessaloniki (Greece) in 2016, 2017, and 2018.

Month	2016			2017			2018		
	T_min_ (°C)	T_max_ (°C)	Rainfall (mm)	T_min_ (°C)	T_max_ (°C)	Rainfall (mm)	T_min_ (°C)	T_max_ (°C)	Rainfall (mm)
April	4	30	8.1	3	26	9.2	6	31	4.9
May	9	32	75.2	11	30	51.8	12	30	93.0
June	14	38	13.3	14	39	13.2	16	33	106.8
July	17	36	0.7	16	40	78.3	17	34	26.1
August	18	36	56.6	14	38	12.9	19	36	12.0
September	9	31	80.0	12	35	2.7	9	34	5.0
Mean	12	34		12	35		13	33	
Total			233.9			168.1			247.8

**Table 3:** Statistical significance (ANOVA) of genotype, growing year, and their interaction on nutritional components, fatty acids composition, phytochemical profile, and antioxidant activity of hemp seeds from the seven industrial hemp cultivars cultivated in this study and for the three growing seasons (2016, 2017, and 2018).

Parameter	Genotype (G)	Total Variance (%)	Year (Y)	Total Variance (%)	GxY	Total Variance (%)
Nutritional Components						
Oil	145.5^***^	58.8	92.5^***^	37.4	9.5^***^	3.9
Protein	38.4^***^	52.4	27.2^***^	37.0	7.8^***^	10.6
Ash	0.1^***^	9.6	0.3^***^	36.9	0.4^***^	53.6
Carbohydrates	30.4.4^***^	57.7	199.0^***^	37.7	24.5^***^	4.6
Fatty Acids						
Palmitic	2.0^*^	86.2	0.1^***^	6.0	0.0^***^	7.8
Stearic	0.3^***^	70.0	0.1^***^	25.7	0.0^NS^	4.3
Oleic	39.0^***^	91.2	2.3^***^	5.5	1.4^***^	3.3
Linoleic	2.8^***^	42.0	3.2^***^	47.7	0.7^***^	10.3
γ-linolenic	3.8^***^	77.3	0.9^***^	18.0	0.2^***^	4.7
α-linolenic	13.0^***^	99.6	0.5^***^	3.3	0.9^***^	4.7
ω-6/ω-3 ratio	1.1^NS^	85.3	0.0^***^	1.3	0.1^***^	13.4
Tocopherols						
γ-Τ	9.1^***^	24.4	26.4^***^	71.0	1.7^***^	4.6
δ-Τ	0.0^***^	9.9	0.2^***^	85.9	0.0^***^	4.2
Carotenoids						
Lutein	1.0^***^	43.7	0.0^***^	12.6	1.0^***^	43.7
β-carotene	0.3^***^	63.5	0.1^***^	28.3	0.0^***^	8.1
Zeaxanthin	0.0^***^	10.1	0.0^***^	64.9	0.0^***^	25.0
Total	2.0^***^	54.3	0.3^***^	7.4	1.4^***^	38.4
Total Phenolic Content	54,456.7^***^	27.3	117,289.3^***^	58.9	27,466.2^***^	13.8
Phenolic Compounds						
Protocatechuic acid	0.3^***^	17.5	1.3^***^	66.6	0.3^***^	15.8
p-hydroxybenzoic acid	0.1^***^	2.9	5.4^***^	93.5	0.2^***^	3.7
Cinnamic acid	1.1^***^	2.9	29.1^***^	77.6	7.4^***^	19.6
N-*trans*-caffeoyltyramine	458.1^***^	6.5	5936.6^***^	84.0	674.7^***^	9.5
Cannabisin A	1738.1^***^	9.7	14,174.2^***^	79.1	1998.5^***^	11.2
Antioxidant Activity						
ABTS assay	105,299.8^***^	26.3	272,547.7^***^	68.1	22,639.5^***^	5.7
FRAP assay	96,613.4^***^	33.7	169,163.2^***^	59.0	20,773.0^***^	7.3

* and *** indicate significance at *p* < 0.05 and 0.001 levels, respectively. ABTS: 2,2′-azino-bis (3-ethylbenzthiazoline sulfonate); FRAP: ferric-reducing antioxidant power; ^NS^: non-significant at *p* > 0.05.

**Table 4:** Fatty acid composition of hemp seeds from the seven industrial hemp cultivars cultivated in this study and for three growing seasons (2016, 2017, and 2018).

Genotype	Year	Fatty Acids (%)						ω-6/ω-3
		Palmitic	Stearic	Oleic	Linoleic	α-Linolenic	γ-Linolenic	
Bialo	2016	8.5 ab	2.4 ab	15.3 b	53.6 a	10.5 b	3.4 a	5.5 a
Felina		8.3 ab	2.4 ab	13.5 c	53.7 a	12.2 a	2.9 b	4.6 c
Tygra		8.2 ab	2.7 a	16.2 ab	51.6 b	11.7 a	2.9 b	4.7 bc
Futura		7.8 b	2.5 ab	16.7 a	52.9 a	10.8 b	2.1 c	5.1 ab
Santhica		8.9 a	2.3 b	12.4 d	54.0 a	12.1 a	3.1 b	4.7 bc
Fedora		-	-	-	-	-	-	-
Finola		-	-	-	-	-	-	-
Bialo	2017	8.6 B	2.6 A	15.8 B	52.7 B	11.4 D	2.6 C	4.9 BC
Felina		7.9 C	2.5 A	13.6 C	53.5 AB	13.0 B	2.6 C	4.4 D
Tygra		8.5 B	2.8 A	17.6 A	51.6 C	10.6 E	2.6 C	5.5 A
Futura		8.5 B	2.8 A	17.9 A	52.9 AB	10.7 E	1.9 D	5.2 A
Santhica		9.1 A	2.5 A	12.6 D	53.9 A	12.1 C	3.0 B	4.7 C
Fedora		8.6 B	2.6 A	15.8 B	52.9 AB	11.2 D	2.9 B	5.0 AB
Finola		7.4 D	2.1 B	10.6 E	53.8 A	15.1 A	4.5 A	3.9 E
Bialo	2018	8.5 **ab**	2.5 **ab**	16.0 **a**	53.5 **b**	11.4 **cd**	2.8 **c**	5.0 **b**
Felina		8.4 **ab**	2.5 **ab**	14.7 **b**	54.2 **a**	11.9 **b**	2.9 **bc**	4.9 **bc**
Tygra		8.3 **b**	2.6 **a**	15.4 **ab**	53.7 **b**	11.7 **bc**	2.9 **bc**	4.8 **bc**
Futura		7.8 **c**	2.7 **a**	16.0 **a**	53.7 **b**	12.1 **b**	3.0 **c**	4.7 **c**
Santhica		8.8 **a**	2.3 **b**	13.2 **c**	54.2 **a**	12.1 **b**	3.2 **b**	4.7 **c**
Fedora		8.7 **ab**	2.6 **a**	15.1 **b**	53.7 **b**	11.2 **d**	3.1 **bc**	5.1 **a**
Finola		7.1 **d**	2.1 **c**	10.3 **d**	53.7 **b**	15.3 **a**	5.0 **a**	3.9 **d**

For each year, means within the column followed by the same letter (lower case, capitals, and lower case in bold for 2016, 2017, and 2018, respectively) are not significantly different (*p* > 0.05). Minor fatty acids such as cis-11-eicosenoic, cis-11,14-eicosadienoic, behenic, arahidic, and lignoceric acids were not evaluated.

**Table 5:** Tocopherols and carotenoid contents (mg 100 g^−1^ seed) of hemp seeds from the seven industrial hemp cultivars cultivated in this study and for three growing seasons (2016, 2017, and 2018).

Genotype	Year	Tocopherols		Carotenoids			
		γ-Τ	δ-Τ	Lutein	β-Carotene	Zeaxanthin	Total
Bialo	2016	8.5 a	0.8 c	2.4 a	0.2 d	0.3 b	2.9 b
Felina		8.6 a	1.0 a	2.1 b	0.6 b	0.3 b	3.0 b
Tygra		7.9 b	0.9 ab	1.8 c	0.5 c	0.3 b	2.6 c
Futura		8.8 a	0.9 b	1.7 c	0.2 d	0.2 d	2.1 d
Santhica		6.4 c	0.8 c	2.3 ab	0.7 a	0.4 a	3.4 a
Fedora		-	-	-	-	-	
Finola		-	-	-	-	-	
Bialo	2017	6.3 D	0.7 BC	0.9 E	0.2 E	0.3 C	1.4 E
Felina		8.0 B	0.8 A	2.4 BC	0.4 CD	0.4 B	3.2 C
Tygra		6.5 CD	0.7 BC	2.0 D	0.6 A	0.4 B	3.0 C
Futura		8.9 A	0.7 BC	2.6 B	0.3 D	0.5 A	3.4 B
Santhica		4.6 E	0.6 C	3.4 A	0.5 B	0.4 B	4.3 A
Fedora		7.0 C	0.8 A	2.2 CD	0.4 C	0.3 C	2.9 CD
Finola		8.4 B	0.8 A	2.2 CD	0.2 E	0.2 D	2.6 D
Bialo	2018	5.2 **cd**	0.6 **c**	2.3 **a**	0.5 **cd**	0.3 **a**	3.1 **a**
Felina		4.8 **d**	0.6 **c**	2.0 **bc**	0.6 **bc**	0.3 **ab**	3.0 **ab**
Tygra		5.6 **c**	0.7 **b**	1.7 **de**	0.8 **a**	0.2 **bc**	2.7 **b**
Futura		6.5 **b**	0.7 **b**	1.5 **e**	0.3 **d**	0.2 **c**	2.0 **c**
Santhica		4.9 **d**	0.6 **c**	2.3 **a**	0.6 **bc**	0.3 **ab**	3.2 **a**
Fedora		5.4 **cd**	0.6 **c**	1.8 **cd**	0.7 **ab**	0.2 **c**	2.8 **b**
Finola		11.3 **a**	1.3 **a**	2.2 **ab**	0.3 **d**	0.4 **a**	2.9 **b**

For each year, means within a column followed by different letters (lower case, capitals, and lower case in bold for 2016, 2017, and 2018, respectively) are significantly different (*p* < 0.05).

## References

[1] Amaducci S., Scordia D., Liu F.H., Zhang Q., Guo H., Testa G.S., Cosentino L. (2015). Key cultivation techniques for hemp in Europe and China. Ind. Crops Prod..

[2] Salentijn E.M.J., Zhang Q., Amaducci S., Yang M., Trindade L.M. (2015). New developments in fiber hemp (*Cannabis sativa* L.) breeding. Ind. Crops Prod..

[3] Andre C.M., Hausman J.F., Guerriero G. (2016). *Cannabis sativa*: The plant of the thousand and one molecules. Front. Plant Sci..

[4] Callaway J.C. (2004). Hemp seed as a nutritional resource: An overview. Euphytica.

[5] Kriese U., Schumann E., Weber W.E., Beyer M., Bruhl L., Matthaus B. (2004). Oil content, tocopherol composition and fatty acid patterns of the seeds of 51 *Cannabis sativa* L. genotypes. Euphytica.

[6] Baldini M., Ferfuia C., Piani B., Sepulcri A., Dorigo G., Zuliani F., Danuso F., Cattivello C. (2018). The performance and potentiality of monoecious hemp (*Cannabis sativa* L.) cultivars as a multipurpose crop. Agronomy.

[7] Fike J. (2016). Industrial hemp: Renewed opportunities for an ancient crop. Crit. Rev. Plant Sci..

[8] Kaul N., Kreml R., Austria A.J., Richard M.N., Edel A.L., Dibrov E., Hirono S., Zettler M.E., Pierce G.N. (2008). A comparison of fish oil, flaxseed oil and hempseed oil supplementation on selected parameters of cardiovascular health in healthy volunteers. J. Am. Coll. Nutr..

[9] Prociuk M.A., Edel A.L., Richard M.N., Gavel N.T., Ander B.P., Dupasquier C.M.C., Pierce G.N. (2008). Cholesterol-induced stimulation of platelet aggregation is prevented by a hempseed-enriched diet. Can. J. Physiol. Pharmacol..

[10] Smeriglio A., Galati E.M., Monforte M.T., Lanuzza F., D’Angelo V., Circosta C. (2016). Polyphenolic compounds and antioxidant activity of cold-pressed seed oil from Finola cultivar of *Cannabis sativa* L.. Phytother. Res..

[11] Pollastroa F., Minassia A., Fresu L.G. (2018). Cannabis phenolics and their bioactivities. Curr. Med. Chem..

[12] Yan X., Tang J., dos Santos Passos C., Nurisso A., Simões-Pires C.A., Ji M., Lou H., Fan P. (2015). Characterization of lignanamides from hemp (*Cannabis sativa* L.) seed and their antioxidant and acetylcholinesterase inhibitory activities. J. Agric. Food Chem..

[13] Legros S., Picault S., Cerruti N., Bouloc P. (2013). Factors affecting the yield of industrial hemp—Experimental results from France. Hemp Industrial Production and Uses.

[14] Blade S.F., Ampong-Nyarko K., Przybylski R. (2005). Fatty acid and tocopherol profiles of industrial hemp cultivars grown in the high latitude prairie region of Canada. J. Ind. Hemp.

[15] Vonapartis E., Aubin M.P., Seguin P., Mustafa A.F., Charron J.B. (2015). Seed composition of ten industrial hemp cultivars approved for production in Canada. J. Food Comp. Anal..

[16] Siano F., Moccia S., Picariello G., Russo G.L., Sorrentino G., Di Stasio M., Cara F.L., Volpe M.G. (2019). Comparative study of chemical, biochemical characteristic and ATR-FTIR analysis of seeds, oil and flour of the edible Fedora cultivar hemp (*Cannabis sativa* L.). Molecules.

[17] Tang K., Struik P., Yin C.X., Thouminot C., Bjelková M., Stramkale V., Amaducci S. (2016). Comparing hemp (*Cannabis sativa* L.) cultivars for dual-purpose production under contrasting environments. Ind. Crops Prod..

[18] European Commission (2014). Commission Regulation (EC) No. 809/2014 of 17 July 2014 laying down rules for the application of Regulation (EU) No 1306/2013 of the European Parliament and of the Council with regard to the integrated administration and control system, rural development measures and cross compliance. Off. J. Eur. Union.

[19] AOAC International (2016). Official Methods of Analysis.

[20] AOAC Method 996-06 (2002). Fats (total, saturated and unsaturated) in foods. AOAC Official Methods of Analysis.

[21] Irakli M., Chatzopoulou P., Kadoglidou K., Tsivelika N. (2016). Optimization and development of a high-performance liquid chromatography method for the simultaneous determination of vitamin E and carotenoids in tomato fruits. J. Sep. Sci..

[22] Singleton V.L., Orthofer R., Lamuela-Raventos R.M. (1999). Analysis of total phenols and other oxidation substrates and antioxidants by means of Folin-Ciocalteu reagents. Methods Enzymol..

[23] Skendi A., Irakli M., Chatzopoulou P. (2017). Analysis of phenolic compounds in Greek plants of Lamiaceae family by HPLC. J. Appl. Res. Med. Arom. Plants.

[24] Re R., Pellegrini N., Proteggente A., Pannala A., Yang M., Rice-Evans C.A. (1999). Antioxidant activity applying an improved ABTS radical cation decolorization assay. Free Radic. Biol. Med..

[25] Benzie F., Strain J. (1999). Ferric reducing/antioxidant power assay: Direct measure of total antioxidant activity of biological fluids and modified version for simultaneous measurement of total antioxidant power and ascorbic acid concentration. Methods Enzymol..

[26] House J.D., Neufeld J., Leson G. (2010). Evaluating the quality of protein from hemp seed (*Cannabis sativa* L.) products through the use of the protein digestibility-corrected amino acid score method. J. Agric. Food Chem..

[27] Galasso I., Russo R., Mapelli S., Ponzoni E., Brambilla I.M., Battelli G., Reggiani R. (2016). Variability in seed traits in a collection of *Cannabis sativa* L. genotypes. Front. Plant Sci..

[28] Kinsella J., Lokesh B., Stone R. (1990). Dietary n-3 polyunsaturated acids and amelioration of cardiovascular disease: Possible mechanisms. Am. J. Clin. Nutr..

[29] Layne K.S., Goh Y.K., Jumpsen J., Ryan R.A., Chow P., Clandinin M.T. (1996). Normal subjects consuming physiological levels of 18:3(n-3) and 20:5(n-3) from flaxseed or fish oils have characteristic differences in plasma lipid and lipoprotein fatty acid levels. J. Nutr..

[30] Da Porto C., Decorti D., Natolino A. (2015). Potential oil yield, fatty acid composition, and oxidation stability of the hempseed oil from four *Cannabis sativa* L. cultivars. J. Diet. Suppl..

[31] Sies H., Murphy M.E. (1991). Role of tocopherols in the protection of biological systems against oxidative damage. J. Photochem. Photobiol. B Biol..

[32] Liang J., Aachary A.A., Thiyam-Holländer U. (2015). Hemp seed oil: Minor components and oil quality. Lipid Technol..

[33] Aladić K., Jokić S., Moslavac T., Tomas S., Vidović J., Vladić S., Šubarić D. (2014). Cold pressing and supercritical CO_2_ extraction of hemp (*Cannabis sativa*) seed oil. Chem. Biochem. Eng..

[34] Okarter N., Liu C., Sorrells M.E., Liu R.H. (2010). Phytochemical content and antioxidant activity of six diverse varieties of whole wheat. Food Chem..

[35] Arts I.C., Hollman P.C. (2005). Polyphenols and disease risk in epidemiologic studies. Am. J. Clin. Nutr..

[36] Teh S.S., Birch J. (2013). Physicochemical and quality characteristics of cold-pressed hemp, flax and canola seed oils. J. Food Comp. Anal..

[37] Chen T., He J., Zhang J., Li X., Zhang H., Hao J., Li L. (2012). The isolation and identification of two compounds with predominant radical scavenging activity in hempseed (seed of *Cannabis sativa* L.). Food Chem..

[38] Pagnani G., Pellegrini M., Galieni A., D’Egidio S., Matteucci F., Ricci A., Stagnari F., Sergi M., Lo Sterzo C., Pisante M. (2018). Plant growth-promoting rhizobacteria (PGPR) in *Cannabis sativa* ‘Finola’ cultivation: An alternative fertilization strategy to improve plant growth and quality characteristics. Ind. Crops Prod..

[39] Heimler D., Vignolini P., Isolani L., Arfaioli P., Ghiselli L., Romani A. (2010). Polyphenol content of modern and old varieties of *Triticum aestivum* L. and *T. durum* Desf. grains in two years of production. J. Agric. Food Chem..

[40] Sakakibara I., Katsuhara T., Ikeya Y., Hayashi K., Mitsuhashi H. (1991). Cannabisin A, an arylnaphthalene lignanamide from fruits of *Cannabis sativa*. Phytochemistry.

[41] Lesma G., Consonni R., Gambaro V., Remuzzi C., Roda G., Silvani A., Vece V., Visconti G.L. (2014). Cannabinoid-free *Cannabis sativa* L. grown in the Po valley: Evaluation of fatty acid profile, antioxidant capacity and metabolic content. Nat. Prod. Res..

[42] Wang C.Z., Ma X.Q., Yang D.H., Guo Z.R., Liu G.R., Zhao G.X., Tang J., Zhang Y.N., Ma M., Cai S.Q. (2010). Production of enterodiol from defatted flax seeds through biotransformation by human intestinal bacteria. BMC Microbiol..

[43] Pojić M., Mišan A., Sakač M., DapčevićHadnađev T., Šarić B., Milovanović I., Hadnađev M. (2014). Characterization of byproducts originating from hemp oil processing. J. Agric. Food Chem..

[44] Frassinetti S., Moccia E., Caltavuturo L., Gabriele M., Longo V., Bellani L., Giorgi G., Giorgetti L. (2018). Nutraceutical potential of hemp (*Cannabis sativa* L.) seeds and sprouts. Food Chem..

[45] Choi Y., Jeong H.-S., Lee J. (2007). Antioxidant activity of methanolic extracts from some grains consumed in Korea. Food Chem..

